# Enrolling adolescents in HIV vaccine trials: reflections on legal complexities from South Africa

**DOI:** 10.1186/1472-6939-8-5

**Published:** 2007-05-13

**Authors:** Catherine Slack, Ann Strode, Theodore Fleischer, Glenda Gray, Chitra Ranchod

**Affiliations:** 1 HIV AIDS Vaccines Ethics Group, School of Psychology, University of KwaZulu-Natal, Private Bag X01, Scottsville, 3209, Pietermaritzburg, South Africa; 2 International Research Ethics Network for Southern Africa; Bioethics Centre, Department of Medicine, Groote Schuur Hospital, Anzia Road, Observatory 7925, Cape Town, South Africa; 3 HIV AIDS Vaccine Division, Perinatal HIV Research Unit, Faculty of Health Sciences, University of the Witwatersrand, PO Box 114, Diepkloof, 1864, Gauteng, South Africa

## Abstract

**Background:**

South Africa is likely to be the first country in the world to host an adolescent HIV vaccine trial. Adolescents may be enrolled in late 2007. In the development and review of adolescent HIV vaccine trial protocols there are many complexities to consider, and much work to be done if these important trials are to become a reality.

**Discussion:**

This article sets out essential requirements for the lawful conduct of adolescent research in South Africa including compliance with consent requirements, child protection laws, and processes for the ethical and regulatory approval of research.

**Summary:**

This article outlines likely complexities for researchers and research ethics committees, including determining that trial interventions meet current risk standards for child research. Explicit recommendations are made for role-players in other jurisdictions who may also be planning such trials. This article concludes with concrete steps for implementing these important trials in South Africa and other jurisdictions, including planning for consent processes; delineating privacy rights; compiling information necessary for ethics committees to assess risks to child participants; training trial site staff to recognize when disclosures trig mandatory reporting response; networking among relevant ethics commitees; and lobbying the National Regulatory Authority for guidance.

## Background

Adolescents have been involved in trials for vaccines to prevent sexually transmitted infections/diseases like Human Papilloma Virus (HPV) and Herpes Simplex Virus type-2, (HSV-2), both in the developed and the developing world [1,2]. In Merck's ongoing quadrivalent HPV vaccine program, at least 12 000 young people between the ages of 9–24 have received the HPV vaccine, with approximately 20% of these being boys and girl between the ages of 9–15 (Merck, personal communication). Infants have also participated in phase I HIV vaccine trials and currently Uganda is enrolling HIV exposed infants in a phase I vaccine trial [3,4]. However, no HIV uninfected adolescents have participated in HIV vaccine trials anywhere in the world.

Because adolescents are severely affected by the HIV epidemic [5-7], they should be the main recipients/beneficiaries of a successful HIV vaccine. To achieve this, there will be a need to license the vaccine for use in this age group. Adolescent participation in HIV vaccine trials is therefore paramount in order to determine the safety profile, appropriate dosing schedules and the degree of immunogenicity in this age group. Adolescent trials are also necessary as vaccine responses may differ because of physiological or hormonal differences between adults and younger adolescents [8,9]. Delays in licensure for use in adolescents could retard the control of the HIV epidemic, as was seen in the control of the Hepatitis B epidemic in the United States where there was no clear strategy to include infants, children and adolescents in the vaccination program [10].

South African researchers anticipate enrolling 16–18 year olds in a phase IIb proof of concept vaccine trial towards the end of 2007. These adolescents will be at high risk of HIV infection. Further, it is envisaged that 12–15 year old adolescents will be involved in phase I/II trials as early as 2008. The phase I/II studies will determine the safety, tolerability and preliminary immunogenicity in pre-teens and young adolescents of candidate vaccines that are currently being tested for preliminary efficacy in adults and older adolescents. The phase I/II studies will involve a small number of healthy adolescents, at low risk of acquiring HIV infection.

In the development and review of adolescent HIV vaccine protocols, there are many legal complexities that need to be addressed. This article sets out complexities linked to consent requirements; special legal protections for children in need of care and protection; and procedural requirements for the approval of such research. These complexities are not unique to South Africa because in many jurisdictions where such trials may occur adolescent participants will have limited legal capacity, and the enrolment of adolescents must take account of local laws dealing with, for e.g., the age of lawful consent to sex and obligations on certain adults to report abuse. Furthermore, like South Africa, very few developing countries will have dedicated research laws. Therefore this article also discusses implications of these legal complexities for role-players in other jurisdictions. We make a series of recommendations for additional work that needs to be done in order to realize the optimal involvement of adolescent participants. We refer to "child" as a person under the age of 18 [11,12] and a "minor" as a person under the age of 21 [13] soon to change to 18 [14]. We use the term "adolescent" to refer to persons between the ages 12 and 18 [15].

### HIV vaccine trials

Phase I/II HIV vaccine trials with adolescents would aim to recruit a small number of healthy adolescents who are at low risk of acquiring HIV infection. Phase IIb trials will recruit adolescents at higher risk of HIV infection. The trials themselves will comprise of a number of interventions, including a general physical examination and medical history-taking; assessment of HIV risk factors including personal questions about sex and substance use; personalized risk reduction counseling; administration of an experimental HIV vaccine or placebo via injection; blood draws for laboratory safety and immunogenicity testing; and regular testing for HIV infection. Adolescents will be classified as "low risk" if they are not sexually active as defined as primary abstinence (no sexual activity ever initiated) or secondary abstinence (no sexual activity for six months). Although many interventions in a phase I trial may not hold out the prospect of direct benefit for adolescent participants, there are interventions that may benefit participants, such as personalised risk reduction counselling. Additionally, there may be associated benefits such as identification of medical conditions like hypertension and early referral to care, access to care for intercurrent illness or reproductive health, and referral for abuse. Despite conceptual difficulties in classifying whole protocols as either "therapeutic" or "non-therapeutic research" [16] it is possible that phase I safety trials in South Africa would be classified as "non-therapeutic" because of the preponderance of interventions that will not confer direct benefit.

## Discussion

### The ethical-legal framework for child research in South Africa

Currently South Africa does not have a comprehensive ethical-legal framework regulating research with children. The Constitution (s12(2) (c)) prohibits research without informed consent [12]. However, there is currently no health or child care legislation dealing directly with child or adolescent research. Health rights are found scattered through the Constitution, common law and various statutes and they do not set out when and how children may participate in research. The primary piece of legislation dealing with the protection of children is the Child Care Act [11] which provides for some of a child's health rights but does not deal with research. In May 2005 the National Health Act [17] became effective. It creates a national framework for health care delivery. Section 71 of the Act covers health research with human subjects including minors. This section has not been implemented as yet and it is unlikely that it will come into effect before the middle of 2007. In June 2006 a new Children's Act [14] was promulgated that will repeal the existing Child Care Act, however, it is not yet in operation. In addition, there are 4 major sets of ethical guidelines in South Africa bearing on child research [18-21]. Recently, draft regulations for research with human subjects were published for public comment [22]. It is envisaged that once the regulations are finalized, s71 will be implemented. Given this state of flux, this article refers to both current and future ethical-legal obligations.

### Key considerations for lawful adolescent HIV vaccine trials

For research to be lawful within any legal system it must comply with substantive and procedural requirements established in law and ethical guidelines. The nature of these obligations varies from system to system, however most establish requirements relating to consent, ethical review and scientific validity. In South Africa, there are three key issues that must be taken into account when ensuring that adolescent research is lawful. These are elaborated on below:

(i) Consent requirements must be met

(ii) Legal obligations in child protection laws must be complied with; and

(iii) There must be compliance with requirements for ethical and regulatory review.

### (i) Informed consent

#### Who must consent?

In South Africa, for adolescent trials to be lawful, consent must given by a participant with legal capacity to consent, or if not competent, by a person with the authority to consent on the participant's behalf.

##### Independent consent by adolescents

In terms of current South African law, there is no provision setting out when children may provide their own independent consent to research. However children may consent independently to medical treatment from the age of 14 [11,23]. Accordingly some legal scholars have argued that children of 14 and older can consent independently to "therapeutic" research [24] while others have argued that such an equation cannot be easily made.^25 ^With regard to "non-therapeutic" research some have argued for independent child consent if there is no risk at all [25] while others have asserted that proxy consent is always required, and can indeed only be given in restricted circumstances [24]. In terms of current South African law, there is also no guidance on who may provide consent for child participation in research if children lack this capacity themselves. It appears that most South African research ethics committees (RECs) have relied on the recommendations made in ethical guidelines which broadly require parental or guardianship consent for research plus the assent of the child [20,21].

##### Consent by a parent or legal guardian and the child if capable of understanding

However under future law, in terms of s 71 of the National Health Act (NHA) [17] consent will have to be obtained from a parent or legal guardian until the age of majority is reached. Other care-givers or custodians will not have the authority to provide consent for child research [23]. Presently, minority ends at 21 [13] however it will soon drop to 18 [14]. While future law will require consent from a sole parent or guardian, some ethical guidelines require consent from both parents (if reasonably available) depending on research risks in relation to direct benefit for the child participant [18,20].

The NHA also specifies that consent must also be obtained from minors if they are "capable of understanding" [17]. There is an implied legal obligation to obtain assent, as assent is the means to get the child's perspective to establish their "best interests" required by s 28(2) of the Constitution for every matter concerning the child [12]. The NHA means that the standard for persons under the age of majority is not necessarily assent but rather that when children have sufficient comprehension it is their consent that shall be required [28]. That is, the Act requires persons who are legal minors to consent rather than assent if they have sufficient comprehension. However, assessment of understanding is potentially complicated [29,30]. While trial sites tend to favour forced-choice checklists to assess understanding because they are objective and easy to administer [31,32], there is some evidence that they may yield higher scores of understanding than open-ended measures like narratives [33] and may be vulnerable to rote memorisation [30,34].

Another complexity is that given that adolescents will not be able to consent to research independently complex privacy issues arise. Although the NHA does not specifically refer to a child/minor's right to privacy in research, a child does have a constitutional and common law right to privacy. In terms of this right a person with an expectation of privacy is entitled to keep aspects of their life private provided this expectation is regarded as reasonable by society [35]. This is also referred to as the "legitimate expectation" of privacy test [36]. In circumstances where a child does not have a right to privacy, researchers may disclose information to the parent or legal guardian as a legitimate party in the research relationship who has provided consent for participation. Applying this test to adolescent research is complex because it is uncertain when society would regard an adolescent's expectation of privacy to be reasonable. *One example*: if an adolescent tests HIV infected during a trial, the adolescent may expect the researchers to keep such information confidential, and society may regard this as reasonable given that adolescent's have the right to HIV testing and confidentiality from the age of 14 outside of a trial context [11]. However, given that parents have provided consent to participation in a trial involving regular HIV testing and given that HIV infected adolescents would require care and support, it may be reasonable to ask adolescent trial participants to waive their privacy rights by agreeing to disclose their HIV status to a trusted adult within a certain time frame.

*A second example*: adolescents may have expectations of privacy regarding their sexual risk information. In this instance society may not regard this as reasonable as parents are legally responsible for their children and are required to protect them from harm. Withholding information from parents regarding risks facing their children such as experimentation with drugs or alcohol may mean that a parent is unable to meet their legal obligations to protect the child. Therefore a parent may be entitled to risk information. However parents could be asked to waive their right to access such information, if other safeguards are in place like referral to counselling, and if the information does not involve significant risks and criminal activity like sexual abuse.

A further complexity is that there is evidence that adolescents when compared to adults are less likely to spontaneously consider risks and benefits, [37] are less likely to evaluate long-term consequences of decisions, [38-40] are more likely to place weight on benefit than risk, [41] are more likely to be short-term focused, [42] and are less likely to recognize the vested interests of others [43]. More risky decisions may be made in groups than individually [44]. Obviously parental consent provides some degree of protection against immature decision-making, however, researchers should try to enhance adolescent understanding to the fullest extent possible.

##### Recommendations

For South African trials, we recommend that HIV vaccine trial researchers anticipate a future change in the age of majority. Researchers could currently consider obtaining parental consent for all under-21's, and be prepared to submit protocol amendments to obtain expedited approval to obtain parental consent for under-18's when the change in the law becomes effective. For trials requiring large numbers of adolescents to be enrolled, researchers must consider how they will assist primary care-givers looking after orphans with the complex legal process of transferring guardianship to enable them to provide lawful consent to adolescent participation. For other jurisdictions, we recommend that clarity be obtained on *whether adolescents can consent independently to research *or if they cannot, *which adults have the capacity to provide proxy consent *byexamining research specific legislation, legislation that establishes the age of majority or that provides adolescents with capacity to consent to specific acts such as medical treatment. Child Care legislation may also describe the persons with legal authority to act on behalf of children. Ethical guidelines should also be consulted for advice.

We recommend that South African researchers consider how they will establish when an adolescent has the necessary depth of understanding for the higher standard of competence required for consent [45] or whether adolescents meet the less strenuous requirements of assent, including basic comprehension of purpose and procedures, and right to withdraw [46] and the ability to indicate a preference [47]. No-one has yet developed an "assent assessment tool" or described what such a tool would assess [48]. As true-false checklists may be inappropriately constituted for the necessary probing required to establish depth of understanding of complex trial concepts [49], sites must consider open-ended measures that probe understanding. Stakeholders outside of South Africa are also likely to be concerned with appropriate "tests" of understanding.

Privacy rights for sexual risk information and HIV status will have to be delineated and both parents and adolescents will have to understand what information parents will/will not have access to. In South Africa this detailed work will hinge on the "legitimate expectation" of privacy test. In other jurisdictions, however, this analysis should also be done using relevant legal principles.

In all settings considering trials, consent processes should be designed that are sensitive to characteristics of adolescent decision-making. While group formats, like Vaccine Discussion Groups, may be effective for disseminating information about trials, cognizance should be taken of how peer influence may affect the evaluation of risk. It is likely that extended interpersonal contact with a knowledgeable trial site counsellor may effectively improve understanding [34] and counsellors should be trained to enhance understanding of potential long-term harms. Adolescent-friendly materials should also be developed with advice from adolescent consultants [50].

#### What can be consented to?

In order for adolescent participation in HIV vaccine trials to be lawful in South Africa, current common law requirements must be met, that is consent to such research must be in accordance with public policy [23]. In other words consent to the harm or risk of harm must be legally permitted [51]. To determine this, it has been proposed that, amongst other factors, the research must present acceptable standards of risk [23]. Neither current South African law nor future law (s71 of the NHA) [17] provides a clear standard for acceptable research risk [23]. Draft regulations [22] assert that research with children is only permissible if it poses minimal risk, or greater than minimal risk but holds out benefit for the child. An important third category is not included in the draft regulations – namely, research that poses more than minimal risk but holds out no prospect of direct benefit. South African ethical guidelines are not unanimous on risk standards however they are approaching harmonisation [52]. Three out of four South African ethical guidelines [18-20] assert that when the intervention or research *does not *hold out the prospect of direct benefit, the allowable risk level is a minor increase over the risks of daily life or routine medical and psychological tests ("everyday risk"); if justified by the risk-knowledge ratio. The one guideline in exception [21] permits no increase at all [53]. Most South African ethical guidelines assert that when the intervention or the research *does *hold out the prospect of benefit there is no explicit upper limit of risk however the risks must be justified by the benefit. It is likely that if HIV vaccine trials are reviewed by a number of RECs, they will disagree on how to apply the risk standards for non-beneficial research [54]. It is possible that different RECs may categorise the same non-beneficial intervention like blood draws for lab testing as minimal risk (permitted), a minor increase over minimal risk (permitted) or more than that (not permitted).

##### Recommendations

Because the majority of recent guidelines now require it, we recommend that RECs become familiar with "component analysis" to demarcate interventions as beneficial or non-beneficial [55] and to assess if non-beneficial interventions meet national risk standards. Investigators should begin now to prepare documentation on risks so that RECs can make these complex assessments for both product related risks and social harms. Data should be available, at least in part, from prior trials with less vulnerable participants such as adults. Inputs should be made to the draft regulations to bring them in line with national ethical guidelines that set acceptable standards of risk for non-beneficial research.

### (ii) Complying with legal obligations in child protection laws

It is a principle of international law that special legal protections ought to exist to protect persons during childhood [56]. South Africa, like most other states therefore have developed a range of special laws that protect children against maltreatment and abuse. For example, in South Africa, the Sexual Offences Act [57] contains specific offences relating to children, such as the criminalization of sex under the age of 16 and child prostitution in s 14(1)(a), 14(3) and 9. Consent is not a defense to these crimes [58]. Furthermore while there is generally no legal obligation to report a crime, South African law includes special protection for children who may be facing abuse, ill-treatment or neglect. The Child Care Act^11 ^in section 42 requires *medical practitioners*, amongst others, to report suspected ill-treatment, abuse or neglect of children to the Department of Social Development. Failure to report is a criminal offence. Additionally, the Family Violence Act [59] states that *any person *who examines, treats, attends to, advises, instructs or cares for any child, who suspects that the child has been ill-treated, must report this to a Commissioner of Child Welfare, a social worker or the police. The future Children's Act [14] in section 150 will oblige *any person *to identify children in need of care and protection (e.g. living in a child headed household, required to perform child labour, being maltreated, abused, or exploited) and to refer these to a social worker. In terms of these laws, it is argued that HIV vaccine trial staff would have a legal duty to report abuse or ill-treatment disclosed by adolescent in a trial. Due to the broad meaning of terms such as "ill-treatment" disclosures of rape and or some cases of under-age sex would need to be reported to the appropriate authorities.

#### Recommendations

We recommend that study staff be trained to recognise those disclosures that trigger a mandatory reporting response. Consent procedures should inform parents and adolescents about this limit to confidentiality. The protocol should not only spell out how formalistic legal requirements will be met but broader ethical requirements to promote children's' welfare, such as whether such information will be disclosed to parents. Furthermore, stakeholders in other jurisdictions will need to establish whether any special protections exist for children, such as the mandatory disclosure of HIV status, and if any special obligations are placed on researchers regarding children in need of care or protection. Such laws may either be found in criminal codes or in child specific laws.

### (III) There is compliance with procedural requirements for ethical and scientific review of the research

To be lawful, research must be approved by the relevant authorities.

#### Ethical approval by an REC

The NHA (s73) [17] sets out the current legal obligations of RECs. It provides that RECs must approve research where it meets the "ethical standards of the committee". It is likely that the MRC (2003) [19] guidelines drafted for HIV vaccine trials will need to be consulted which allow child participation provided risk standards, consent and scientific necessity requirements are met.

#### Authorisation for the use of a genetically modified organism

In terms of current law, for all HIV vaccine trials, a permit must be obtained from the Executive Council of Genetically Modified Organisms for any research into a genetically modified organism such as an HIV vaccine. This is a body established within the Department of Agriculture [60].

#### Approval by the Medicines Control Council (MCC)

In terms of current law [61], the Minister of Health in consultation with the MCC has the power to issue regulations on the control and conduct of clinical trials. These have been issued and provide (amongst other things) that clinical trials must be conducted in accordance with Good Clinical Practice guidelines issued by the MCC or the Department of Health from time to time [62]. While the MCC [63] has also prepared a set of guidelines for phase I trial applications they do not appear to have issued any guidance on adolescents.

#### Ministerial consent for "non-therapeutic" research

Phase I adolescent HIV vaccine trials may be classed as "non-therapeutic". When Section 71(3)(b)(iv) of the NHA [17] becomes effective, "non-therapeutic" research on children may not be done without first obtaining consent from the Minister of Health. S/he may not consent to such research if amongst others it poses a "significant risk" to the health of a child, or "some" risk that is not outweighed by benefit. No definition is provided of "significant risk", and this is not provided for in the draft regulations [22]. This condition adds an additional layer of administrative scrutiny [23,27]. However, much remains to be clarified, including which research falls into its scope, its place in the sequence of approvals investigators must obtain, and the entity that will carry out this function. This detail is also not provided in the draft regulations [22].

#### Recommendations

We recommend that RECs begin to network with each other to build consensus about adolescent trials, including the acceptability of trial interventions in terms of national risk standards. Like regulatory authorities in all the jurisdictions planning adolescent HIV vaccine trials, the Medicines Control Council should be requested to articulate the data it will require to *firstly*, allow adolescents into trials and *secondly*, to license an adolescent vaccine. Very specifically, in the South African setting, we recommend that researchers anticipate the public policy assessment that the Minister will have to undertake by framing their protocols in a way that assists the Minister, or delegated authority, to make a speedy determination. RECs in all jurisdictions planning such trials should be aware that public policy considerations are becoming increasingly important in the regulation of research and are being reflected in law. Therefore research-specific and health specific laws should be consulted to establish whether there are specific limits on certain forms of research. Researchers who craft their protocols with thoughtful attention to ethical guidelines may meet most, if not all, of the legal requirements. Where the law is unclear, researchers should consult with their REC or get legal advice from a lawyer trained in research ethics and law.

## Conclusion

HIV vaccine trials with adolescents will pose legal complexities for all jurisdictions in which they will take place. Complexities may stem from a lack of legal guidance, or a lack of tools for using legal concepts, and some disharmony between ethical guidelines [22]. The legal complexities are not, however, insurmountable. Stakeholders preparing for these trials in South Africa and other countries should take on the following as a matter of urgency:

1. Investigators must plan for the complex consent processes that will be required including assessment of understanding

2. Investigators must compile the information necessary for RECs to assess potential risks to child participants (both social and physical) to establish if these meet national risk standards for child research

3. It must be determined whether adolescents have privacy rights to their sexual risk information and HIV status and if so, whether these will be waived or not

4. Trial site staff must be trained to recognize when adolescent disclosures (e.g. of abuse or neglect) will trigger a mandatory reporting response

5. RECs that will review adolescent protocols should begin to network with one another to prepare for a coordinated response to similar research protocols, and

6. The national regulatory authority should outline the data they will need to allow adolescent trials and allow licensure of an adolescent vaccine.

*A journey of a 1000 miles starts with a single step*.

## Summary

Enrolling adolescents in HIV vaccine trials will pose legal complexities in all jurisdictions where these will occur, likely beginning with South Africa. Investigators and REC will have to deal with i) consent requirements (e.g. who must consent? what can be consented to?); ii) obligations to protect children from abuse and maltreatment (e.g. responding to disclosures by adolescents that they have been raped, or are having unlawful sex) and iii) procedural requirements for approval of the research (e.g. need for guidance from the National Regulatory Authority). Jurisdictions planning adolescent HIV vaccine trials, like South Africa, will have to consider a range of networking, tool development and training processes to ensure that sound adolescent trials are a reality.

## Competing interests

The author(s) declare that they have no competing interests.

## Authors' contributions

Ms Slack and Strode conceived of the legal analysis and prepared the preliminary analysis. Dr Fleischer redrafted sections of the legal analysis and helped to draft the overall manuscript. Dr Gray prepared the introductory sections, made inputs to the legal analysis and helped to draft the overall manuscript. Ms Ranchod helped to prepare the analysis of the consent requirements and assisted with drafting the overall manuscript. All authors read and approved the final manuscript.

## Pre-publication history

The pre-publication history for this paper can be accessed here:


